# Fermentation of lactose to ethanol in cheese whey permeate and concentrated permeate by engineered *Escherichia coli*

**DOI:** 10.1186/s12896-017-0369-y

**Published:** 2017-06-02

**Authors:** Lorenzo Pasotti, Susanna Zucca, Michela Casanova, Giuseppina Micoli, Maria Gabriella Cusella De Angelis, Paolo Magni

**Affiliations:** 1 Laboratory of Bioinformatics, Mathematical Modelling and Synthetic Biology, Department of Electrical, Computer and Biomedical Engineering, University of Pavia, via Ferrata 5, 27100 Pavia, Italy; 2 Centre for Health Technologies, University of Pavia, 27100 Pavia, Italy; 3 Centro di Ricerche Ambientali, IRCCS Fondazione Salvatore Maugeri, via Salvatore Maugeri 10, 27100 Pavia, Italy

**Keywords:** Ethanol, Lactose, Fermentation, *Escherichia coli*, Whey permeate

## Abstract

**Background:**

Whey permeate is a lactose-rich effluent remaining after protein extraction from milk-resulting cheese whey, an abundant dairy waste. The lactose to ethanol fermentation can complete whey valorization chain by decreasing dairy waste polluting potential, due to its nutritional load, and producing a biofuel from renewable source at the same time. Wild type and engineered microorganisms have been proposed as fermentation biocatalysts. However, they present different drawbacks (e.g., nutritional supplements requirement, high transcriptional demand of recombinant genes, precise oxygen level, and substrate inhibition) which limit the industrial attractiveness of such conversion process. In this work, we aim to engineer a new bacterial biocatalyst, specific for dairy waste fermentation.

**Results:**

We metabolically engineered eight *Escherichia coli* strains via a new expression plasmid with the pyruvate-to-ethanol conversion genes, and we carried out the selection of the best strain among the candidates, in terms of growth in permeate, lactose consumption and ethanol formation. We finally showed that the selected engineered microbe (W strain) is able to efficiently ferment permeate and concentrated permeate, without nutritional supplements, in pH-controlled bioreactor. In the conditions tested in this work, the selected biocatalyst could complete the fermentation of permeate and concentrated permeate in about 50 and 85 h on average, producing up to 17 and 40 g/l of ethanol, respectively.

**Conclusions:**

To our knowledge, this is the first report showing efficient ethanol production from the lactose contained in whey permeate with engineered *E. coli*. The selected strain is amenable to further metabolic optimization and represents an advance towards efficient biofuel production from industrial waste stream.

**Electronic supplementary material:**

The online version of this article (doi:10.1186/s12896-017-0369-y) contains supplementary material, which is available to authorized users.

## Background

Whey is an abundant waste stream generated during cheese production. After cheese curdling, about 10% of the used milk is converted in cheese, while the remaining liquid is a by-product called whey, which still contains about 55% of the milk nutritional load [1, 2]. In particular, although whey composition depends on several factors (e.g., milk quality, animal breed and feed), a high lactose concentration (about 45 g/l), and about 6–10 g/l of proteins are usually present [3–6]. They correspond to the total amount of milk lactose content and about 20% of milk proteins, respectively [2].

Worldwide, 160 million tons of whey per year are produced [2]. It represents an environmental problem for its high nutritional load, largely due to lactose content, and, for this reason, it cannot be discharged in water systems without pre-treatments [3]. Several options, extensively reviewed by Prazeres et al. [4], are available to decrease the organic content of whey and, in some cases, valorize this waste to obtain added-value bioproducts at the same time: biological treatments with or without valorization, physicochemical treatment and direct land application. Valorization can be carried out by recovering high-value biomolecules, such as the protein fraction that is separated via ultrafiltration or diafiltration, obtaining whey protein concentrates which can be used in food, cosmetic and pharmaceutical industries. The liquid remaining after this process (called whey permeate - WP) has the same lactose concentration as whey and for this reason its pollution load is still high [4]. Furthermore, WP can be concentrated to facilitate its transportation to treatment plants, obtaining concentrated whey permeate (CWP) that can have a lactose concentration up to about 160 g/l. Extraction of lactose from whey or WP is not always economically convenient. Hence, lactose to ethanol fermentation is considered as a further treatment of WP, required to decrease its pollutant load, simultaneously enabling the production of a commercially attractive biomolecule from waste material [2, 4, 7]. Ethanol can be used as a fuel, but also in food and beverages, pharmaceutical and cosmetic industries.

In large-scale production plants, ethanol is normally obtained from sugars (e.g. sucrose molasses and hydrolyzed starch) by alcoholic fermentation of the baker’s yeast *Saccharomyces cerevisiae* or the soil bacterium *Zymomonas mobilis*, which are not able to ferment lactose [2, 8]. Enzymatic or chemical pre-hydrolysis of lactose into glucose and galactose and subsequent feeding of *S. cerevisiae* with these sugars can be carried out [9], but it is not economically convenient for the high cost of the required enzymes or chemical pre-process [2, 10] and for the catabolite repression phenomenon [11]. Strains affected by such phenomenon show slower fermentations of sugar mixtures, such as glucose and galactose, compared to strains without catabolite repression, although mutant yeasts not exhibiting this phenotype can be selected [12]. Naturally occurring organisms able to ferment lactose into ethanol include *Torula cremoris*, *Kluyveromyces fragilis*, *Kluyveromyces lactis*, *Kluyveromyces marxianus* and *Candida pseudotropicalis* yeasts [2]. However, despite examples of industrial implementation of whey-to-ethanol production plants using whey or deproteinated whey as substrate are present [2], such microorganisms are not ideal workhorses for the ethanol production industry because several drawbacks affect their use in large-scale plants, such as low productivity, metabolic inhibition at high lactose concentration, complex nutritional requirements and impaired growth in low-oxygen conditions [2]. Because of such drawbacks, research is still ongoing to optimize the underlying conversion processes and test different naturally-occurring strains as biocatalysts [2, 5, 13, 14]. For the above reasons, whey-to-ethanol fermentation technology requires a dramatic improvement in order to enhance the attractiveness of the bioprocess [2, 7].

Engineered microorganisms able to convert lactose to ethanol have been constructed. The main examples include metabolic modifications of ethanol-producing baker’s yeast to enable lactose fermentation, and of lactose-consuming bacteria to enable ethanol production from pyruvate. Since this work focuses on the latter, particularly on *Escherichia coli*, the main literature outcomes are briefly reported, while the efforts to create metabolically engineered *S. cerevisiae* are reviewed elsewhere [2]. Wild type *E. coli* is able to ferment lactose to produce a mix of organic acids and a low amount of ethanol [15]. Efforts have been carried out in the last 30 years to build recombinant *E. coli* expressing the *Z. mobilis* pdc and adhB genes (encoding pyruvate decarboxylase and alcohol dehydrogenase, respectively) to direct pyruvate metabolism towards ethanol production, by the pyruvate to acetaldehyde and carbon dioxide reaction via pdc, and the acetaldehyde to ethanol reaction via adhB. The two ethanologenic genes have been assembled with their native ribosome binding sites (RBSs) in an operon and expressed under the control of different promoters via multicopy plasmids [16–18] or integrated in the genome [19] of strains previously tested for their environmental hardiness and substrate fermentation range [18], with the final aim of constructing a biocatalyst for efficient fermentation of plant biomass sugars [20–22]. The strains constructed in literature often showed high transcriptional demand for pdc and adhB, which needed very strong promoters, multicopy plasmids or multiple tandem chromosomal repeats to be properly expressed [19, 23–25]. For instance, KO11, the first constructed and most widely used *E. coli* strain with pdc and adhB in the chromosome [19], has about 25 copies of the ethanologenic genes [26], generated via antibiotic selection procedure to obtain high ethanol production, which was low in the initial clone. The transcriptional demand may also affect the genetic stability of the genes [15, 20, 27, 28], and represents a problem when dealing with scenarios typical of real industrial settings, like the use of poor media, in which nutrients can be insufficient for suitable expression of ethanologenic genes [23, 29]. Expensive nutrient supplementations could be avoided in minimal media by using other strains that have been proposed by re-engineering existing biocatalysts using random integration sites and selection based on growth and ethanol production [30] or re-engineering different hosts [24]. Strains were also engineered for algal biomass fermentation [31]. Despite no *E. coli* strain was specifically engineered to ferment dairy waste, examples of successful ethanol production from cheese whey via the KO11 strain have been reported and nutrient supplementation was needed to obtain a reasonable fermentation performance [32, 33]. Finally, engineered strains have also been constructed, again mainly focused on cellulosic biomass fermentation, using different strategies, such as laboratory metabolic evolution [34], rational pathway engineering supported by elementary mode analysis [35], stabilization of plasmid-borne pdc-adhB via mutations that can be complemented only via ethanologenic genes in strict anaerobic growth [36, 37], and genome engineering without foreign genes [38, 39].

In summary, a number of studies have reported the successful construction and characterization of ethanologenic *E. coli* strains, but no specific study has been carried out to construct an ad-hoc strain for dairy waste fermentation. The studies above shed light on many critical issues that can support the engineering of future ethanologenic microbes. In this work, we aim to metabolically engineer a set of candidate *Escherichia coli* strains (reported in Table 1) via a new pdc-adhB expression plasmid, and we carry out the selection of the best strain in terms of growth in permeate, lactose consumption and ethanol formation. We finally show that the selected engineered microbe is able to efficiently ferment WP and CWP, without nutritional supplements, in pH-controlled bioreactor.

**Table 1:** Candidate host strains for lactose to ethanol fermentation and expression plasmids used in this study

Strain denomination	Code	Source
Strains		
B	DSM 613	Deutsche Sammlung von Mikroorganismen und Zellkulturen GmbH (DSMZ)
B/r	DSM 500	Deutsche Sammlung von Mikroorganismen und Zellkulturen GmbH (DSMZ)
C	DSM 4860	Deutsche Sammlung von Mikroorganismen und Zellkulturen GmbH (DSMZ)
W	DSM 1116	Deutsche Sammlung von Mikroorganismen und Zellkulturen GmbH (DSMZ)
ML308	DSM 1329	Deutsche Sammlung von Mikroorganismen und Zellkulturen GmbH (DSMZ)
Crooks	ATCC 8739	American Type Culture Collection (ATCC)
MG1655 (K-12)	CGSC 7740	Coli Genetic Stock Center (CGSC, Yale University)
W3110 (K-12)	CGSC 4474	Coli Genetic Stock Center (CGSC, Yale University)
Plasmids		
pL13	pSB4C5 with BBa_K173022 as insert	This study
pLOI297	ATCC 68239	American Type Culture Collection (ATCC)

It is worth noting that the search of optimal fermentation parameters is not addressed in this work. An exploratory analysis is herein carried out by testing a number of experimental conditions, which represent only a subset of all the possible ones, to test WP and CWP fermentation feasibility. A systematic parameter search for the selected strain will be investigated in future studies.

## Results

### pL13 construction and preliminary characterization in LB

The pL13 expression plasmid was designed and constructed to meet the following specifications: an operon structure with adhB-pdc driven by the regulated Plux promoter (BBa_R0062 from the Registry of Standard Biological Parts) [40]; strong RBSs (BBa_B0030 from the Registry of Standard Biological Parts) [41] assembled upstream of both genes; a low-copy number replication origin, enabling plasmid maintenance at 3–7 copies per cell [42, 43]. Together with the codon-optimized design of pdc and adhB, the above specifications aim to maximize the translational efficiency of the two recombinant genes, to reduce the high transcriptional demand reported in the literature for previously developed strains. Although the quantitative levels of gene expression and protein synthesis are hard to predict in operon architecture and many context-dependent features can affect them [44], software tools are available to support their forward and reverse engineering. A recently proposed tool for sequence-to-function prediction, based on biophysical model of translational coupling (Operon Calculator [45]), estimated a 10- and 90-fold improvement in translation initiation rate of pdc and adhB, respectively, in our synthetic operon compared to the operon integrated in the KO11 genome [26]. Finally, in our operon, the Plux promoter also enables downstream mRNA expression tuning over a wide range of transcriptional activities to probe optimal enzyme levels if required [40]. In this work, only the Plux basic activity was exploited for pdc and adhB expression.

**Fig. 1:**
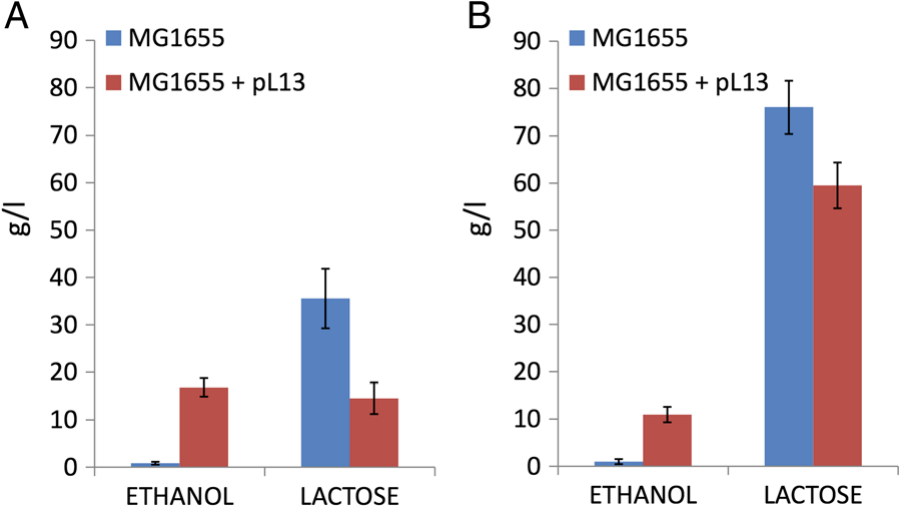
Preliminary fermentation experiments with wild type and engineered MG1655 in LB with phosphate buffer at the indicated lactose concentration in 15-ml tubes. **a** 40 g/l of lactose. **b** 80 g/l of lactose. Ethanol and residual lactose are measured after a 72-h fermentation at 30 °C. The reported values are the mean of at least 4 independent replicates and error bars are the 95% confidence intervals of the mean

The pL13 plasmid was initially transformed in MG1655, a widely used K-12 strain, to perform preliminary experiments. Enzymatic assays for pdc and adhB showed a successful expression of both enzymes (Table 2). Fermentation experiments in LB supplemented with 40 g/l of lactose in 15-ml tubes demonstrated that the engineered MG1655 could produce high levels of ethanol (17 g/l) in 72 h at 30 °C, consuming more than half of the available lactose (Fig. 1a). The non-engineered strain, conversely, produced less than 1 g/l of ethanol and consumed a much lower amount of lactose. The engineered MG1655 also showed typical traits of ethanologenic strains [16]: higher pH (6 versus 5, despite the presence of phosphate buffer) and cell concentration (OD600 values of ~1.0 versus ~0.5, see Methods section for details about cell density measurements) at the end of fermentation, compared to the non-engineered strain. The higher pH is the result of the decrease of organic acid production fluxes from pyruvate, caused by the recombinant pathway introducing a pyruvate to ethanol route. At the reached concentrations, ethanol is less toxic than the organic acids produced in the wild type fermentation pathway [17] and the recombinant cultures could reach a higher cell density than the non-engineered MG1655. Fermentation experiments in LB + 80 g/l of lactose confirmed the ethanol production performance (Fig. 1b), pH (6 versus 5) and growth (~1.0 versus ~0.6) of MG1655 with pL13. Although these preliminary experiments demonstrated the successful functioning of the recombinant pathway, they highlighted an incomplete lactose consumption, probably due to the lack of a strict pH control, which can inhibit cell growth and ethanol production [46].

**Table 2:** Relative enzymatic activity of pdc and adhB in four different engineered strains and standard error of the mean value (in brackets) for at least two independent measurements

Engineered strain	Relative pdc activity^a^	Relative adhB activity^a^
MG1655-pL13	0.22 (0.005)	0.23 (0.124)
ML308-pL13	1 (-)	1 (-)
W-pL13	0.156 (0.012)	0.17 (0.019)
W-pLOI297	1.26 (0.325)	2.32 (0.831)

^a^All the reported activities for both pdc and adhB are normalized by the ones of ML308-pL13, measured in the same experiment. For this reason, the activity values of ML308-pL13 are always 1 and no standard error can be computed. Wild type strains were also assayed as controls and they showed no detectable activity for both enzymes (data not shown)

### Strain selection

After MG1655 engineering, the pL13 plasmid was incorporated into other seven *E. coli* strains (Table 1), which were then tested in WP in terms of growth, lactose consumption and ethanol formation to select the best biocatalyst for fermentation. The eight strains of Table 1 were selected to meet these main specifications for an efficient biocatalyst: non-pathogenic strains able to consume lactose, possibly used previously for ethanol production from different substrates (meaning that the strain is amenable for genetic modifications and may be also suitable for ethanol production from dairy waste) [18, 19, 24, 30, 31, 35, 38, 39] and possibly wild type strains (suggesting that they can display a fast-growth phenotype without auxotrophies, compared with highly engineered laboratory strains). All the regulatory parts of the pL13 expression plasmid, i.e., promoter, RBSs and low-copy replication origin, were tested as previously reported [47, 48] and they resulted to be fully functional in the eight candidate strains (data not shown).

**Fig. 2:**
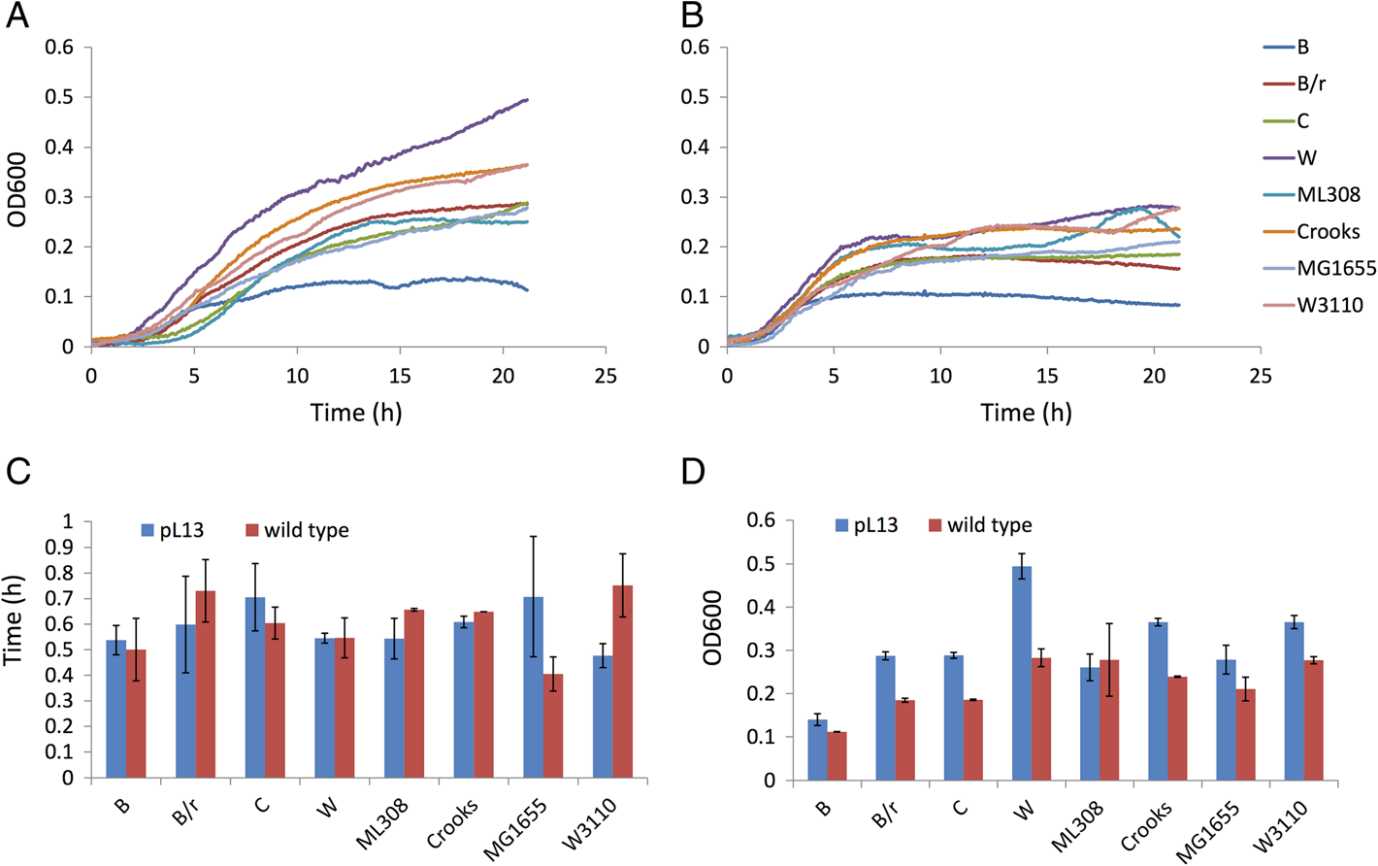
Growth curves in WP for the eight candidate strains in 96-well microplates experiments. **a** Growth assay results for the strains engineered with pL13. **b** Growth assay results for the non-engineered strains. **c** Doubling time in exponential growth phase. **d** Maximum OD600 reached in the experiment. Data points in panels **a**-**b** and bars in panels **c**-**d** represent the average values of at least two independent replicates. Error bars in panels **c**-**d** represent the 95% confidence intervals of the mean (due to the presence of an outlier, exhibiting noisy measurements in the exponential growth phase, no replicates were available to compute confidence intervals for the wild-type Crooks strain in panel **c**)

Growth assays in 96-well microplates demonstrated that all the strains could successfully grow in WP (Fig. 2a-b). Doubling times in exponential growth phase exhibited a narrow range (0.4–0.75 h, considering average data), and did not show relevant differences between engineered and non-engineered strains (Fig. 2c). The maximum cell density reached in a 21-h growth time showed that engineered strains could reach higher cell densities (1.4-fold on average) than the wild types, as expected (Fig. 2d). Moreover, maximum cell density spanned a 5-fold range (0.11–0.49, considering average data) among different strain backgrounds, with the W strain showing the highest OD600, followed by the Crooks and W3110 strains. The B strain showed the lowest OD600.

**Fig. 3:**
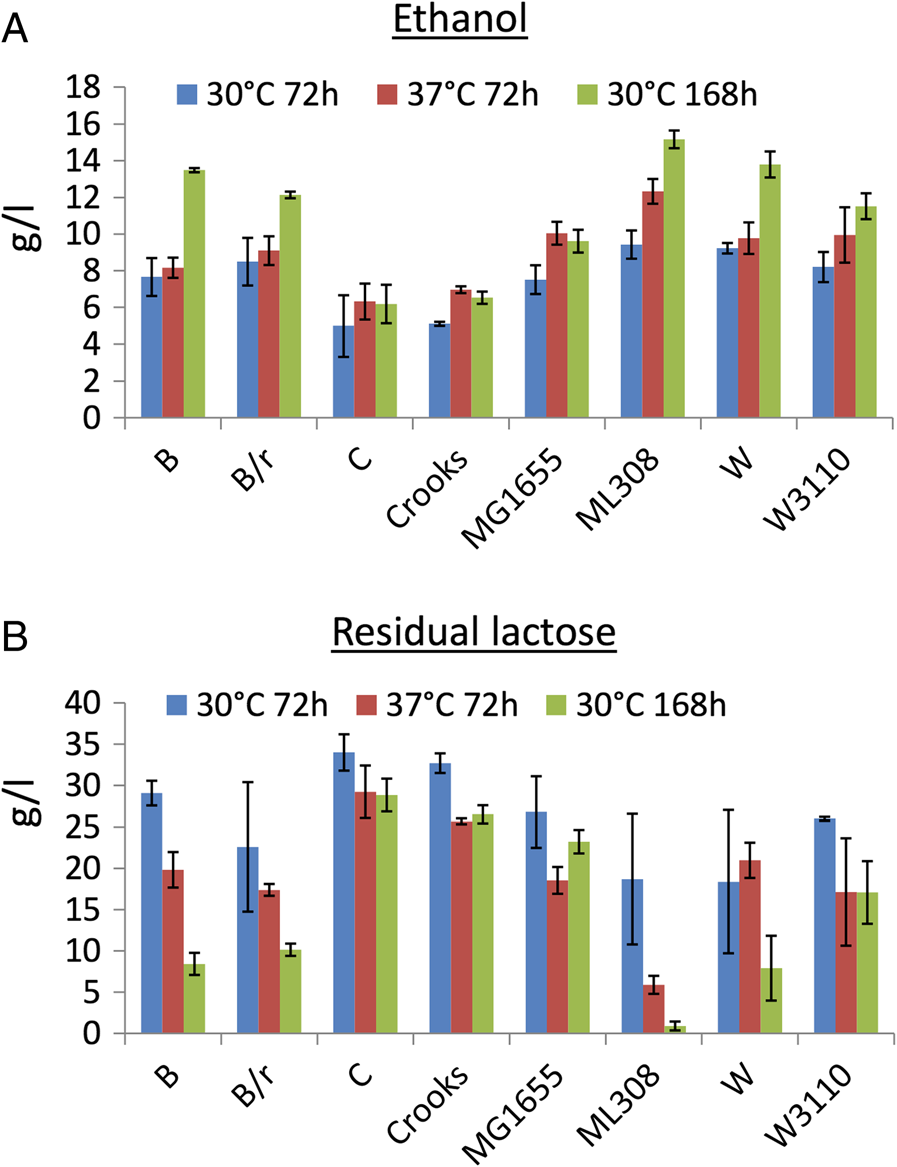
Fermentation results in WP for the eight candidate strains with pL13 in 15-ml tubes. **a** Ethanol production. **b** Residual lactose. Bars represent the average values of 2 to 4 independent experiments. Error bars represent the 95% confidence intervals of the mean. The initial lactose concentration of the used WP batch was about 45 g/l

Fermentation experiments in 15-ml tubes, carried out in WP at two temperatures (30 °C, measured at 72 h and 168 h, and 37 °C, measured at 72 h, see Fig. 3), showed that all the engineered strains could successfully produce ethanol from lactose. The ML308 strain, followed by the W strain, gave the best performance, while the C and Crooks strains had the lowest one. In particular, 15.2 g/l of ethanol were produced by the ML308 strain in 168 h, with a residual lactose of less than 1 g/l, while 13.8 g/l of ethanol were produced by the W strain in the same conditions, with less than 8 g/l of residual lactose. Data after 72 h also confirmed that ML308 and W were superior, with about 9 g/l of ethanol and 18 g/l of residual lactose for both strains. Enzymatic activities were also measured for these two strains (Table 2). As expected, a correlation was present between pdc and adhB activities in each strain with pL13 (MG1655, W and ML308), since the two recombinant genes are assembled in the same operon. A >5-fold activity range was observed in the three different strain backgrounds for both enzymes (0.156–1 for pdc and 0.17–1 for adhB), confirming that they were properly expressed and also supporting previous findings that the same expression plasmid for pdc and adhB could give highly different enzyme activities in different hosts [18].

Considering all strains after 72 h, the mean ethanol concentration was slightly higher at 37 °C than at 30 °C (9.1 versus 7.6 g/l; *p*-value < 0.05, paired *t*-test).

The maximum ethanol concentration reached by the Crooks and C strains in the best condition (37 °C at 72 h) was 2-fold lower than the one reached by the two strains exhibiting superior performance (W and ML308), and a considerably high residual lactose was also left (>25 g/l). Among the other strains, it is worth noting that the B strain also showed high ethanol production (13.5 g/l) and low residual lactose (8.4 g/l) after 168 h, but its performance after 72 h was poor, probably for its poor growth capability in WP (see Fig. 2a). This strain was already used as a host for ethanol production with promising results [18, 39], also from lactose [18], but the results shown here highlight the need for a specific screening for ethanol production and sugar consumption in dairy waste. The Crooks strain was also used in literature as an efficient ethanol producer from different sugars (not lactose), but we could not observe reasonably good performance in the conditions tested here (see Fig. 3), despite its promising results in terms of growth capability (see Fig. 2d).

Considering the growth and fermentation results, the W and ML308 strains were selected for further study.

### Fermentation of permeate and concentrated permeate in pH-controlled bioreactor

**Fig. 4:**
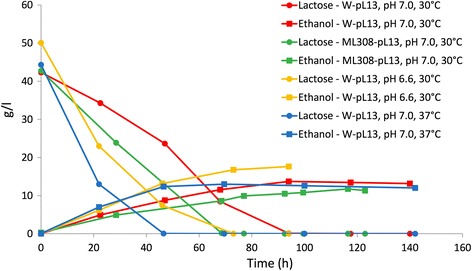
Fermentation of WP in a pH-controlled bioreactor. Ethanol and lactose concentrations over time are shown for different conditions in terms of temperature (30 °C or 37 °C), pH (6.6 or 7.0) and host strain (W or ML308) bearing pL13

The parallelized assays carried out so far did not enable the characterization of fermentation performance in conditions compatible with industrial settings, in which pH control is essential to maximize bioconversion efficiency parameters, such as maximum product concentration, fermentation yield and volumetric productivity [46]. In a preliminary pH-controlled bioreactor experiment for the W strain carried out in LB + 80 g/l of lactose at 30 °C, pH 7.0, we observed a high ethanol production (29 g/l) after only 1 day, with a complete lactose consumption (Additional file 1: Figure S1), corresponding to 70% of the maximum theoretical yield (the main fermentation parameters, including the ones mentioned above, are shown in Table 3 for each experiment of this study). We then carried out pH-controlled exploratory experiments in WP to evaluate the fermentation feasibility and performance in different pH, temperature and engineered strain contexts (Fig. 4). In the tested conditions, we found that the engineered W strain could consume all the lactose and convert it into ethanol with a 54–65% conversion yield. The ML308 strain, tested at 30 °C, pH 7.0, showed slightly lower conversion yield and productivity than the W strain in the same conditions (51% versus 60% and 0.13 versus 0.15 g/l/h). According to the data in Fig. 4 and Table 3, we selected the 37 °C, pH 6.6 condition for further investigation, despite a slightly lower fermentation yield than in the test at 30 °C, already observed in other ethanologenic *E. coli* with different fermentation substrates [46]. In particular, the fast conversion obtained at 37 °C (also highlighted by the 2-fold increase in volumetric productivity from the test at 30 °C to the one at 37 °C, both at pH 7.0, see Table 3) is attractive in industrial context, since a decrease in process time can result in a more rapid turnover of the fermentation tanks, even if the increased energy demand and slight yield decrease should be considered. One of the conditions above (W strain, 37 °C, pH 7.0) was also scaled-up by 8-fold in a 2.4-liter volume without performing filter-sterilization of WP. Results showed analogous fermentation time, maximum ethanol concentration and productivity to the small-scale experiment (Fig. 4 and Additional file 1: Figure S2). Although the lactose to ethanol conversion yield in WP was lower than the one observed in a rich medium like LB (70%, see Additional file 1: Figure S1 and Table 3), these results are promising and a complete lactose consumption was always observed, producing 11.7 to 17.6 g/l of ethanol in the tested conditions.

**Table 3:** Fermentation performance parameters in all the pH-controlled experiments carried out in this work

Engineered strain	Fermentation medium	pH	Temperature (°C)	Maximum ethanol concentration (g/l)	Fermentation time (h)	Fermentation yield (% of theoretical maximum yield)	Initial lactose (g/l)	Volumetric productivity (g/l/h)
W-pL13	LB	7.0	30 °C	28.9	25	70	76.5	1.1
W-pL13	WP	7.0	30 °C	13.7	94	60	42.3	0.15
W-pL13	WP	6.6	30 °C	17.6	73	65	50.1	0.23
W-pL13	WP	7.0	37 °C	13.0	46.5	54	44.3	0.27
W-pL13	WP^a^	7.0	37 °C	12.8	50	45	52.4	0.26
W-pL13	CWP	6.6	37 °C	35.2	71.5	56	116.6	0.46
W-pL13	CWP	6.6	37 °C	33.3	71	45	137.4	0.42
W-pL13	CWP	6.6	37 °C	40.5	116	64	117.4	0.33
ML308-pL13	WP	7.0	30 °C	11.7	68.5	51	42.7	0.13
ML308-pL13	CWP	6.6	37 °C	33.3	42.5	39	158.3	0.72
ML308-pL13	CWP	6.6	37 °C	35.9	74	62	106.3	0.44
W-pLOI297	CWP^b^	6.6	37 °C	37.8	71	63	110.7	0.52

^a^in a 2.4-liter volume without filter-sterilization of the medium

^b^ 0.8 μm filter-sterilization of the medium

**Fig. 5:**
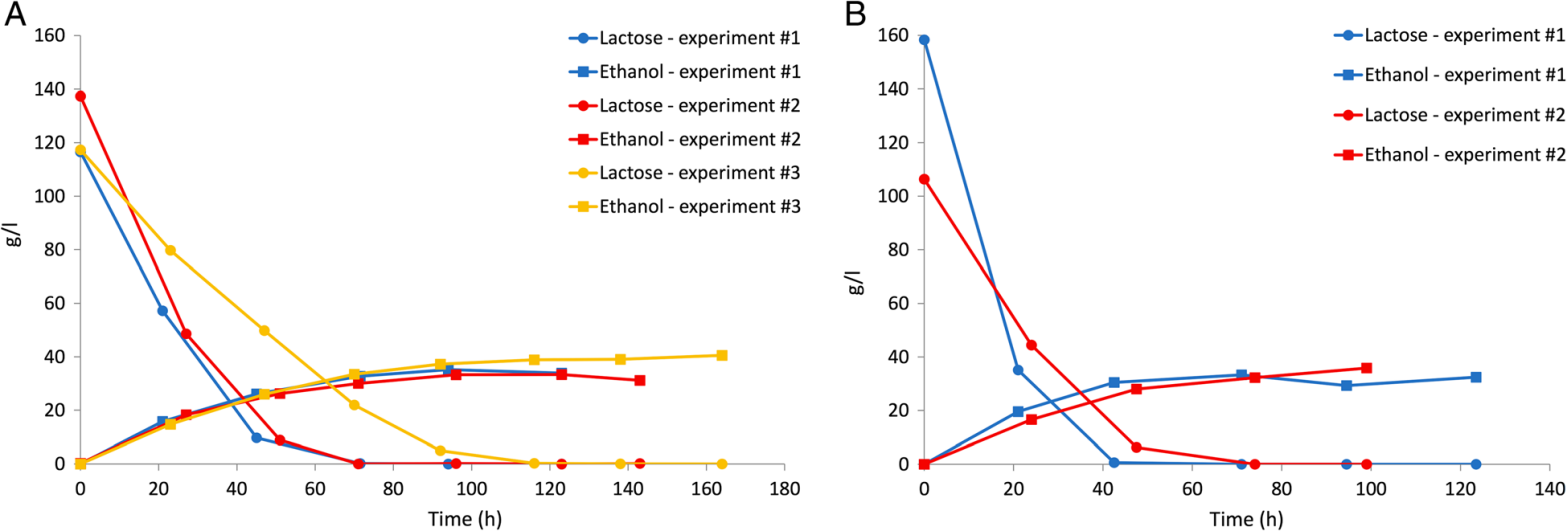
Fermentation of CWP in a pH-controlled bioreactor for W-pL13 (**a**) and ML308-pL13 (**b**). Ethanol and lactose concentrations over time are shown for three (**a**) or two (**b**) independent experiments carried out at 37 °C, pH 6.6

We finally carried out three fermentation experiments for the engineered W strain in CWP (Fig. 5a). The use of a waste with a higher lactose concentration than WP is industrially attractive from an economical point of view, since a higher amount of ethanol will be present in the fermentation broth and distillation costs per liter of ethanol will decrease. However, the possible additional costs of the concentration process should also be considered. Results show that the engineered W strain can also efficiently ferment CWP with a 45–64% conversion yield, producing up to 40 g/l of ethanol and consuming all the lactose (117 to 137 g/l according to the three waste batches) in about 70 (two replicates) to 116 (one replicate) h. These data demonstrate that, although conversion yield could be further improved and a relevant variability is displayed, the lactose to ethanol fermentation in WP and CWP is feasible via an ad-hoc selected engineered bacterial strain without nutritional supplements. The engineered ML308 strain was also tested in the same conditions and the results of two experiments (Fig. 5b) showed performance comparable with the engineered W strain in terms of conversion yield (39–62%) and maximum ethanol concentration (up to 36 g/l). Despite a relatively low conversion yield was observed in the first experiment with ML308 (39%), the measured volumetric productivity (0.72 g/l/h) was almost 2-fold higher than in the other replicate and in the experiments with the W strain. By comparing the average productivity and conversion yield between the two strains, no statistically significant difference was found (*p*-value > 0.05, *t*-test).

The W strain was also transformed with pLOI297 (ATCC 68239, prepared according to ATCC instructions), a high-copy number plasmid for the high-level expression of wild-type pdc-adhB, used for efficient ethanol production in different published works [18, 35, 46]. The resulting engineered strain (W-pLOI297) was tested in CWP. Results showed a comparable performance with our W-pL13 strain (Table 3 and Additional file 1: Figure S3), although the activity of pdc and adhB was much higher with pLOI297 than with pL13 (Table 2). Such data suggest that the basic activity of the Plux promoter in pL13 is sufficient to drive the expression of pdc and adhB at suitable levels for ethanol production in WP and CWP, and the use of a plasmid providing higher activity levels of both enzymes does not seem to result in a clear improvement of fermentation performance.

## Discussion

In this work, a new engineered biocatalyst has been constructed, with design features specific for ethanol production from dairy waste. To our knowledge, this is the first report showing ethanol production from the WP and CWP lactose with engineered *E. coli*. The main steps of this work included the construction of a new expression plasmid, strain selection in parallelized experiments in WP and CWP, and pH-controlled fermentation tests. The new plasmid (pL13) includes a fully synthetic operon for pdc and adhB expression, engineered to maximize the translation of the two proteins. Translation was maximized to overcome the high transcriptional demand previously observed for wild-type ethanologenic genes that often needed to be expressed at high levels or placed at high DNA copy numbers, often via tandem chromosomal repeats amplification, to enable sufficient ethanol production [19, 23, 25, 26], and for these reasons also resulting in unstable systems [20, 27]. The maximization of translation was carried out via codon optimization and use of efficient RBSs. The basic activity of a regulated promoter, Plux, in the off-state was used to drive the expression of pdc and adhB in pL13. We planned to use such promoter to produce a versatile plasmid for pdc and adhB expression tuning via chemical inducer (N-3-oxohexanoyl-L-homoserine lactone), but we found that the basic transcriptional activity already gave reasonably good performance in the engineered W and ML308 strains. For this reason, no inducer was added in this work. The fermentation performance of W-pLOI297 was comparable to the one of our W-pL13 strain, supporting the assumption that the pdc and adhB levels produced by pL13 are sufficient for an efficient ethanol production in the considered dairy waste. Nonetheless, their activity is much lower in pL13 than in pLOI297 and previous works reported correlation between enzyme activity and fermentation yield [17, 23]. While the major aim of this work was to demonstrate the feasibility of WP and CWP fermentation via ad-hoc selected engineered *E. coli*, further work should be carried out on our new codon-optimized genes to understand their optimal expression level and try to further improve ethanol yield.

Strain selection process resulted in the choice of the W and ML308 strains as best engineered biocatalysts, according to their growth and fermentation performance. Although these two strains were already considered as efficient ethanol production systems, their choice among the candidate strains was not trivially predictable. In fact, other strains that have been extensively used in literature as efficient biocatalysts were excluded: the B strain showed poor growth in WP and high ethanol levels only after 168 h, while the Crooks strain showed high level growth but low ethanol production. These results further demonstrate the need of strain selection for the specific task and process to be carried out.

Tests in pH-controlled bioreactor were carried out considering both W and ML308 strains, which showed comparable performance in terms of fermentation profiles and parameters. However, we identified the engineered W strain as the best candidate between the two strains for two main reasons: i) it is more amenable to further metabolic engineering steps [49], since its genome is fully available [50]; ii) its transformation efficiency is much higher than ML308, which was the only strain among the ones in Table 1 that showed poor efficiency (data not shown), also by using a different transformation protocol [51].

The lactose to ethanol conversion efficiency did not considerably vary among the experiments carried out in dairy waste in different conditions. However, it is worth noting that several problems might affect fermentation performance in industrial context, such as waste composition and contaminations. In our experiments, initial concentration of lactose showed variability in both WP and CWP (see Table 3). Given a condition, fermentation efficiency itself can vary, as shown in the experiments with two or three replicates in CWP. Additional studies concerning fermentation (e.g., pH and temperature tuning) and bioprocess (e.g., inoculum size and preparation) optimization will be needed to demonstrate not only the feasibility of CWP fermentation via engineered *E. coli*, but also its economic competitiveness compared with other processes with different biocatalysts. One preliminary test was successfully carried out in this work using a 2.4-liter culture without sterilization of WP, thus providing promising results concerning contamination problems. When the optimal enzyme levels, fermentation parameters and bioprocess steps are defined, a proper number of experimental replicates and long-term operation times will provide useful insights into the real sustainability of the process. In this work, a number of conditions was tested, but a systematic search of optimal fermentation parameters was not addressed and additional investigations will be performed. Here, only an exploratory analysis was carried out, testing a subset of all the possible conditions, to support strain selection (in 15-ml tubes and 96-multiwell plates experiments) and to test the feasibility of WP and CWP fermentation (in pH-controlled experiments). In particular, strain growth was assayed in WP in 96-multiwell plates via OD600 measurements; ethanol and lactose concentrations were measured in 15-ml tubes fermentations at two temperatures after 72 h, and only one of the conditions was also tested after 168 h to evaluate if prolonged fermentation time could improve fermentation performance. Analogously, pH-controlled fermentations in WP were carried out in three temperature/pH conditions without testing a full factorial design, and the parameter values associated with improved fermentation performance were chosen for the other tests.

## Conclusions

A new engineered biocatalyst was constructed, with design features specific for ethanol production from dairy waste. To our knowledge, this is the first report showing ethanol production from WP and CWP by engineered *E. coli*. The strain, selected by a growth/fermentation screening assay, is amenable for further genetic modifications, e.g., gene knockout and heterologous gene expression optimization, to disrupt competing pathways and improve lactose-to- ethanol flux. Our biocatalyst could efficiently ferment two dairy waste streams, derived from an existing valorization chain, without nutritional supplements, providing promising results towards the green, sustainable and economically attractive conversion of such waste into a biofuel.

## Methods

### Strains and growth media

TOP10 (Invitrogen) *E. coli* were used for cloning according to manufacturer’s instructions. Bacterial hosts for ethanol production are described in Table 1. All of them were cultured at 37 °C in LB medium (10 g/l NaCl, 10 g/l bactotryptone, 5 g/l yeast extract, and 15 g/l of agar if preparing LB agar plates) following the instructions provided by DSM, ATCC or CGSC. A glycerol stock, routinely stored at -80 °C, was prepared for each strain by mixing 250 μl of sterile 80% glycerol with 750 μl of bacterial culture.

For lactose-supplemented LB, lactose (L2643, Sigma Aldrich) was dissolved in deionized water, filter-sterilized (0.2 μm), and added to autoclaved LB to reach a final concentration of 40 or 80 g/l. When required, 100 mM of phosphate buffer at pH 7.0 was added [46].

WP and CWP were retrieved from the Recetto (Italy) whey processing plant (Negri Alimenti company, Italy). WP comes from a whey protein concentrates extraction process via ultrafiltration. CWP is the result of either a reverse osmosis process on WP, or a ultrafiltration process on whey that has been previously concentrated by reverse osmosis. They were stored in a refrigerator in the plant and transported in non-refrigerated conditions. After delivery, the batches used in this work had an initial lactose concentration and a pH of 42–52 g/l and 6.5–6.7 (WP), and 106–158 g/l and 6.0–6.2 (CWP), respectively. Glucose and galactose, also measured in preliminary tests, typically accounted for only 2% of the total sugars in WP and CWP. Unless differently stated, WP and CWP were stored at -20 °C and filter-sterilized (0.2 μm) before use. When required, 200 mM of piperazine-N,N’-bis(2-ethanesulfonic acid) (PIPES) buffer at pH 7.0 was added. Antibiotics were always added to maintain plasmids in engineered strains during cloning and fermentation experiments: ampicillin (100 mg/l), kanamycin (50 mg/l) or chloramphenicol (12.5 mg/l).

### Cloning

The *pdc* and *adhB* gene sequences were designed with the Mr Gene GmbH (Germany) codon-optimization service and obtained via *de-novo* DNA synthesis. Their sequences were submitted to the MIT Registry of Standard Biological Parts (Registry, http://partsregistry.org) open source archive as BBa_K173016 and BBa_K173017 entries. All the other DNA parts were retrieved from the iGEM 2008 and 2009 DNA Distributions. The pL13 plasmid was assembled via BioBrick Standard Assembly as previously described [42], using the pSB4C5 low-copy number vector backbone. Its construction process and final sequence can be accessed in the BBa_K173022 entry of the Registry. Unless differently stated, competent cells for the candidate strains were prepared as follows: 5 ml of LB were inoculated with glycerol stock of the desired strain and incubated overnight at 37 °C, 220 rpm; the culture was 250-fold diluted in 50 ml of LB in a flask and incubated in the same conditions as above until it reached an OD600 of 0.14 (relative to 200 μl of culture in a 96-well microplate measured via an Infinite F200 reader - Tecan); the culture was chilled in ice and centrifuged (4000 rpm, 4 °C, 15 min); the supernatant was removed and the pellet was resuspended with 30 ml of an MgCl2 (80 mM) + CaCl2 (20 mM) buffer; cells were centrifuged as before and the pellet was resuspended with 2 ml of a CaCl2 (100 mM) + glycerol (1.5%) buffer; cells were transferred into 0.5 ml tubes and stored at -80 °C before use. Bacterial transformation was carried out by heat shock at 42 °C to obtain the recombinant strains.

### Growth assays in 96-well microplate

Two to five ml of LB medium were inoculated with the glycerol stocks of the non-engineered and engineered strains. Cultures were incubated overnight at 30 °C, 220 rpm. These cultures were 100-fold diluted in a 96-well microplate in 200 μl of filter-sterilized (0.2 μm) WP, which had been stored at +4 °C for 24 h after its delivery. WP without bacteria was also added to the microplate wells. The microplate was incubated at 30 °C for 21 h in the Infinite F200 (Tecan) reader, with the following kinetic cycle, programmed via the i-control software (Tecan) [52]: linear shaking for 15 s at 3-mm, wait for 5 s, absorbance measurement at 600 nm, repeat every 5 min. At least two independent replicates were analyzed.

### Fermentation experiments in 15-ml tubes

Two ml of LB + 40 g/l lactose were inoculated with the glycerol stocks of the strains. Cultures were incubated overnight at 30 °C or 37 °C, 220 rpm. These cultures were centrifuged, their supernatant was removed, the pellet was resuspended in 9 ml of WP + PIPES, and incubated in the same conditions as above for 72 or 168 h. Finally, the cultures were centrifuged, the supernatant was filter-sterilized (0.2 μm) and stored at -20 °C before HPLC analysis. Fermentations using LB + phosphate buffer and 40 g/l or 80 g/l lactose were carried out analogously (at 30 °C, 72 h), with the exceptions that the 2 ml culture was in LB + phosphate buffer + 40 g/l lactose and it was 100-fold diluted in 9 ml of fermentation medium.

### Fermentation experiments in pH-controlled bioreactor

Seventy ml of LB + 40 g/l lactose were inoculated with the glycerol stocks of the strains. Cultures were incubated overnight at 30 °C or 37 °C, 220 rpm. These cultures were centrifuged (4000 rpm, 10 min), their supernatant was removed, the pellet was resuspended in 300 ml of fermentation medium, and incubated in the Minifor (Lambda) laboratory-scale bioreactor (0.4-liter vessel) at the same temperature as above, pH 6.6 or 7.0 and agitation set to 4.0 (arbitrary units). The pH was controlled via addition of KOH 3 M, actuated via the PRECIFLOW peristaltic pump (Lambda). Fermentation in 2.4 l culture volume was carried out as above, except that the pre-inoculum was done in 500 ml of LB + 40 g/l lactose, the 7-liter vessel was used, and agitation was set to 6.0 (arbitrary units). Fermentation in LB + 80 g/l lactose was carried out as above, except that the pre-inoculum was done in 3 ml of LB + 40 g/l lactose, and the grown culture was used to inoculate 300 ml of LB + 80 g/l lactose as fermentation medium. Fermentation was carried out in a sterile setup, following the manufacturer recommendations. Gas was allowed to escape from the vessel, to avoid excessive pressure, via a silicon tube connected with a 0.2 μm filter and put into a flask filled with water to limit ethanol loss due to evaporation. The ethanol loss in the described fermentation setup was experimentally demonstrated to be negligible (see Additional file 1: Figure S4).

### Quantitative measurements of fermentation performance

Lactose and ethanol concentrations were measured via a Shimadzu 10 AD/vp HPLC system equipped with a Supelco C-610H 30 cm x 7.8 mm column (59320-U, Sigma Aldrich) and a RID 10A detector (Shimadzu). The column was kept at 30 °C. H_3_PO_4_ 0.1% was used as mobile phase at the flow rate of 0.5 ml/min. The injection of 25 μl was carried out via automatic injector. The pdc and adhB activities were measured via specific enzymatic assays as previously described [53], except that the results were normalized by the activity in the ML308 strain, which was included in each experiment, to decrease the variability of the assay in different reaction mix batches.

### Data analysis

In microplate growth assays, for each time point the raw absorbance of WP was subtracted from the absorbance of wells with bacterial cultures to yield the optical density at 600 nm time series of the bacterial cells (OD600, which is proportional to cell density). Doubling time was computed as described by Mandell et al. [54]. One-sided t-tests were performed with Microsoft Excel.

The LabSolutions software (Shimadzu) was used to analyze HPLC data. Lactose and ethanol concentrations were used to compute the fermentation yield as 100*(g/l of ethanol)/(0.54*g/l of lactose), where 0.54 is the theoretical maximum ethanol yield that can be obtained from lactose [46]. Fermentation time (t_f_) was reported as the time in which residual lactose is less than 1% of the initial lactose. Volumetric productivity was computed as the ethanol concentration at t = t_f_ divided by t_f_.

## Additional file

Additional file 1: Figure S1. Fermentation of LB supplemented with 80 g/l of lactose in a pH-controlled bioreactor for W-pL13. **Figure S2.** Fermentation of WP without filter-sterilization in a pH-controlled bioreactor for W-pL13 in a 2.4-liter culture. **Figure S3.** Fermentation of CWP in a pH-controlled bioreactor for W-pLOI297. **Figure S4.** Evaluation of ethanol evaporation in a bioreactor experiment. (PDF 352 kb)

## Abbreviations

ATCC: American type culture collection
CGSC: Coli genetic stock center
CWP: Concentrated whey permeate
DSMZ: Deutsche Sammlung von Mikroorganismen und Zellkulturen GmbH
HPLC: High-performance liquid chromatography
OD600: Optical density at 600 nm
RBS: Ribosome binding site
RID: Refraction index detector
WP: Whey permeate
