# Supplementary material for: Fermentation of lactose to ethanol in cheese whey permeate and concentrated permeate by engineered Escherichia coli
Source: BMC Biotechnol. 2017 Jun 2;17:48. doi: 10.1186/s12896-017-0369-y (PMC5457738; doi:10.1186/s12896-017-0369-y)

Additional file

Supplementary Figure 1. Fermentation of LB supplemented with 80 g/l of lactose in a pH-controlled bioreactor for W-pL13. Ethanol and lactose concentrations over time are shown in an experiment carried out at 30°C, pH 7.0.

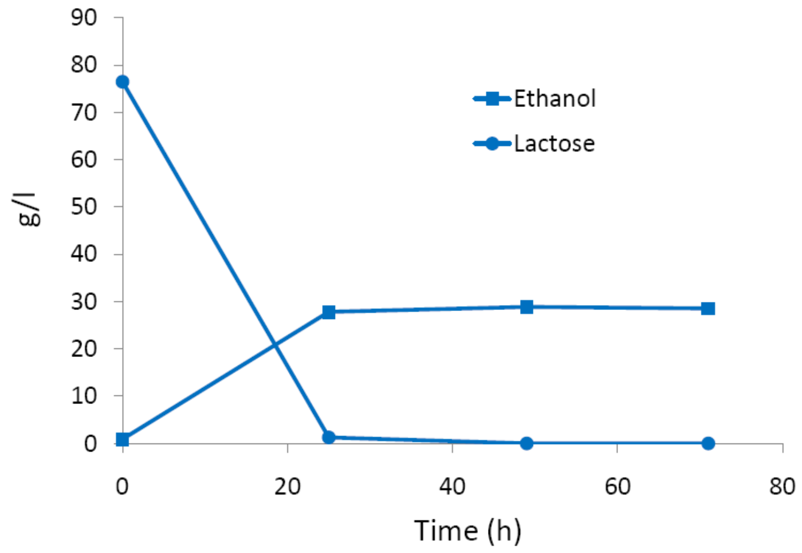

Supplementary Figure 2. Fermentation of WP without filter-sterilization in a pH-controlled bioreactor for W-pL13 in a 2.4-liter culture. Ethanol and lactose concentrations over time are shown for experiment carried out at 37°C, pH 7.0.

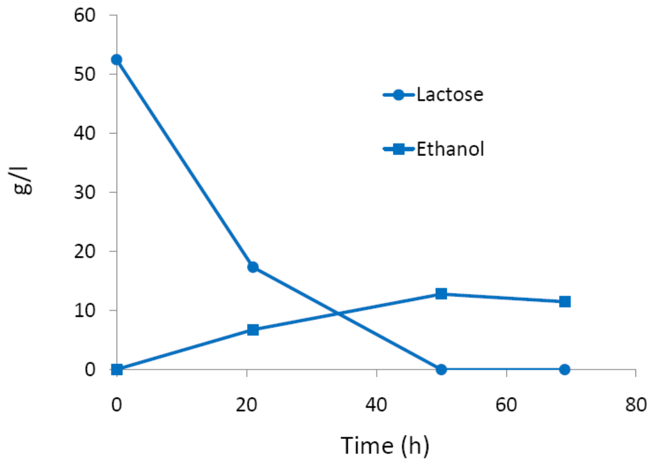

Supplementary Figure 3. Fermentation of CWP in a pH-controlled bioreactor for W-pLOI297. Ethanol and lactose concentrations over time are shown for an experiment carried out at 37°C, pH 6.6.

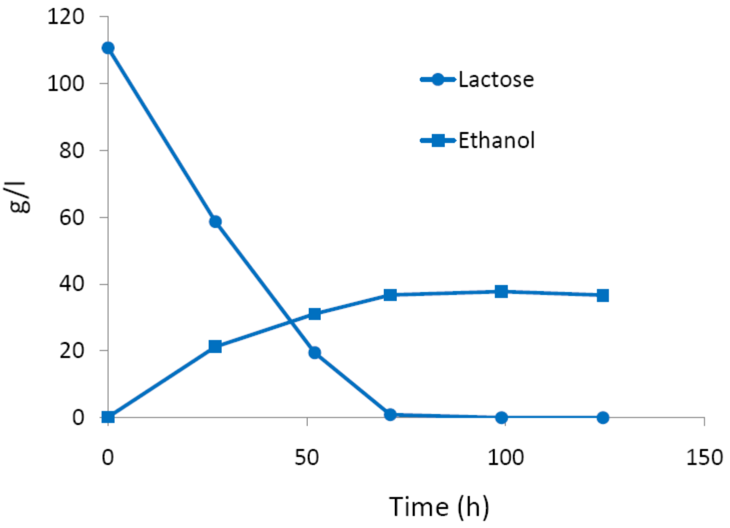

Supplementary Figure 4. Evaluation of ethanol evaporation in a bioreactor experiment. Ethanol concentration was measured at different time points in a control experiment carried out with the same fermentation setup adopted in the other experiments, in CWP at 37°C, pH 6.6, without inoculating cells, and adding 50 g/l of ethanol at t=0. No relevant ethanol loss was detected (less than 5% of the initially measured concentration after about 70 h).

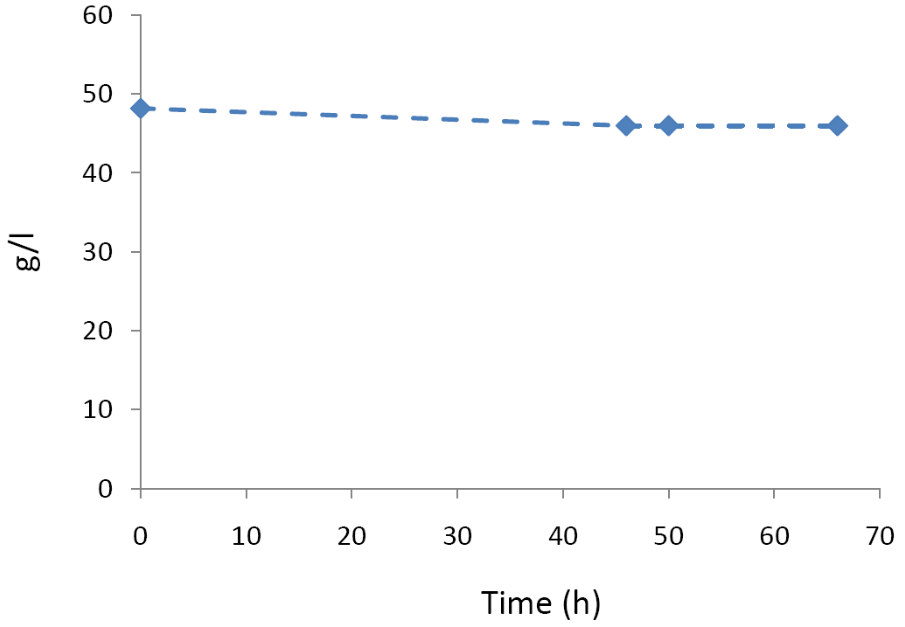

Supplement: Additional file 1: Figure S1. — Fermentation of LB supplemented with 80 g/l of lactose in a pH-controlled bioreactor for W-pL13. Figure S2. Fermentation of WP without filter-sterilization in a pH-controlled bioreactor for W-pL13 in a 2.4-liter culture. Figure S3. Fermentation of CWP in a pH-controlled bioreactor for W-pLOI297. Figure S4. Evaluation of ethanol evaporation in a bioreactor experiment. (PDF 352 kb) [file 12896_2017_369_MOESM1_ESM.pdf]
